# Online Media Platform and Continuous Positive Airway Pressure Machine Purchasing Prior to Bariatric Surgery: Qualitative Study

**DOI:** 10.2196/84098

**Published:** 2026-04-15

**Authors:** Bundit Sawunyavisuth, Darunee Jongudomkarn, Kittisak Sawanyawisuth

**Affiliations:** 1 Department of Marketing, Faculty of Business Administration and Accountancy, Khon Kaen University, Khon Kaen, Thailand; 2 Department of Family and Community Nursing, Faculty of Nursing, Khon Kaen University, Khon Kaen, Thailand; 3 Department of Medicine, Faculty of Medicine, Khon Kaen University, 123 Mitraparp road, Khon Kaen, 40002, Thailand, 66 43363664

**Keywords:** continuous positive airway pressure, bariatric surgery, social media, online information, weight reduction, support group, obstructive sleep apnea, CPAP, stigma

## Abstract

**Background:**

Bariatric surgery offers quick weight reduction for patients with morbid obesity. Those who plan for bariatric surgery require perioperative preparation, including obstructive sleep apnea (OSA) evaluation, and treatment using a continuous positive airway pressure (CPAP) machine is recommended. There are limited data on how patients have prepared for bariatric surgery or for those who have decided to purchase a CPAP machine prior to surgery.

**Objective:**

This study aimed to use a qualitative method to evaluate how obese patients with OSA who were scheduled for bariatric surgery received information regarding bariatric surgery, including their decision to purchase a CPAP machine prior to bariatric surgery.

**Methods:**

This qualitative study enrolled adult patients who planned to undergo bariatric surgery, were diagnosed with OSA, and were treated with CPAP therapy at least 1 month prior to surgery. An in-depth interview was conducted with eligible patients, addressing their perspectives on obesity, strategies for weight loss, and reasons for purchasing or renting CPAP machines prior to bariatric surgery. The interview was conducted using content analysis and triangulation focused on a variety of data informants, theoretical sampling, and achieving data saturation. Themes were summarized and reported.

**Results:**

There were 14 patients with obesity and OSA who planned for bariatric surgery. The average age of all patients was 27.21 (SD 4.98) years with male proportion of 28.57% (4/14) and single marital status of 78.57% (11/14). The average BMI was 45.28 (SD 7.58) kg/m^2^ and the average apnea-hypopnea index was 40.42 (SD 29.61) events per hour. Seven themes were reported: the causes of obesity, effects of obesity, effects of weight loss, experiences of social media on weight loss, CPAP therapy prior to bariatric surgery, and experiences of social media on bariatric surgery. Morbidly obese patients with OSA who planned for bariatric surgery experienced physical and mental effects of obesity, including social stigmatization. These patients had failed in various weight loss programs and believed that bariatric surgery was the correct solution. Social media was used for data gathering in terms of bariatric surgery and CPAP therapy. CPAP rental or secondhand purchasing was preferred.

**Conclusions:**

Social media platforms are a source of information prior to bariatric surgery. A CPAP machine is not a lifesaving machine; rather, it is a temporary treatment before surgery and may not be required after weight loss. CPAP rental or secondhand purchasing may be preferred. These are participant beliefs, not clinical conclusions.

## Introduction

Obstructive sleep apnea (OSA) and obesity are global public health issues and are closely related [1]. One study showed that approximately 936 million people worldwide were affected with OSA [2], while more than 1 billion people were overweight or obese in 2022 [3]. Both diseases are associated with cardiovascular diseases, such as hypertension or left ventricular hypertrophy [4,5]. A 10% weight gain increases the risk of apnea by 32%, while weight loss lowers the risk by 26% [6]. One study showed that after 10 years, a 7.1-kg weight loss resulted in an OSA remission rate of 34.4% [6].

Bariatric surgery is a quick method of weight reduction for patients with morbid obesity. This is indicated if the BMI is ≥35 kg/m^2^, or BMI of 30‐34.9 kg/m^2^ with metabolic disease [7]. Weight loss by bariatric surgery may vary and depend on gender and procedures. Women had a total weight loss of 3.1% more than men, and the Roux-en-Y procedure resulted in 6.9% more weight loss than sleeve gastrectomy [8]. Additionally, bariatric surgery cured OSA in 54% (38/70) of patients, while 20% (14/70) still had moderate-to-severe OSA 5 years after surgery [9].

Preoperative preparation is crucial and includes a psychological and nutritional assessment, weight loss plan, and medical clearance [10,11]. Two previous studies found that although preoperative evaluation and preparation are essential, they were still lacking from both the patients’ and health care providers’ perspectives [12,13]. These issues included education materials, accessible sources, resource needs, and support groups. One Canadian study found that better resources for bariatric surgery are needed from the perspective of health care providers, while patients also reported that there is unmet information regarding bariatric surgery. As 1 patient mentioned, “I don’t want to bother the doctor.” Additionally, a continuous positive airway pressure (CPAP) machine is needed for several weeks prior to the surgery to prevent postoperative complications [11].

A systematic review found that patients with OSA are at risk for complications after bariatric surgery, with a relative risk of 1.23; *P*=.04 [14]. If treated with CPAP preoperatively, the major complications of bariatric surgery were comparable with those patients without OSA [15]. Despite the benefits of CPAP treatment preoperatively, the adherence was only 45% [16]. Some patients with OSA who planned to undergo bariatric surgery may not have purchased a CPAP machine for personal use, but such evidence is limited. Therefore, this study used a qualitative method to evaluate how obese patients with OSA who were scheduled for bariatric surgery received information regarding bariatric surgery, including their decision to purchase a CPAP machine prior to bariatric surgery.

## Methods

### Study Setting

This study was conducted at Khon Kaen University. OSA was diagnosed by the presence of an apnea-hypopnea index (AHI) of 5 or more events per hour using polysomnography. A CPAP machine was available at the hospital, online, or rented from CPAP representatives. The study was conducted between October 2023 and February 2024.

### Study Design

This qualitative research, based on Husserlian descriptive phenomenology, studies the lived experiences of obese patients seeking weight loss through gastric surgery. The patients were required to prepare for bariatric surgery by using a CPAP machine for 1 month prior to the procedure. The research presentation follows the COREQ (Consolidated Criteria for Reporting Qualitative Research) 32-item checklist [17].

### Researcher Positioning and Reflexivity

The research team consisted of 3 people, all holding doctoral degrees: 1 in marketing, 1 in nursing, and 1 in medicine. A research assistant, also with a doctorate in nursing, was responsible for collecting all qualitative data. Both the researchers and the research assistant were experienced and had undergone training and practical experience in qualitative interview research. No prior relationship was established with participants before the study. To minimize potential bias, the researcher maintained a reflexive journal to reflect on assumptions, positionality, and the influence of professional background during data collection and analysis. The research team emphasized bracketing to “switch off” biases and preconceived notions, in order to embrace the true experience of the informants, unadulterated by the researchers themselves.

### Study Setting and Context

Thailand is a Southeast Asian country in which most of the population practices Buddhism. This study was conducted in Northeastern Thailand, locally known as Isan, a region with a population of approximately 21.7 million people, accounting for nearly one-third of Thailand’s total population. Isan comprises 20 provinces across northern, central, and southern subregions and is predominantly rural and agricultural in nature.

Theravada Buddhism is the primary religion in Isan and strongly influences social values and everyday life. Its emphasis on the Buddhist middle path encourages emotional restraint and acceptance, alongside an optimistic worldview. These religious and cultural orientations shape interpersonal relationships and approaches for coping with life challenges.

Data collection took place in a public tertiary-level hospital, which provides advanced medical services and serves as a referral center for patients from both urban and rural areas. All study participants resided in the Isan region.

Isan society is characterized by strong familial and community ties, with cultural values emphasizing kinship, mutual assistance, and interdependence among family members. Extended family living arrangements are common, often involving 3 to 4 generations within the same household. There is limited cultural expectation for younger family members to live independently, and family interdependence typically continues across the life course, including after marriage.

Although urbanization and social change have influenced lifestyles—particularly among younger generations—core Isan cultural values remain prominent. A shared belief that individual suffering is collectively experienced by the family underpins caregiving practices and reciprocal support within households. These contextual and cultural characteristics form an important backdrop for understanding participants’ experiences in this study [18,19].

### Participants and Sampling

Participants were recruited using convenience sampling from patients scheduled for bariatric surgery who were using a CPAP machine and met the study inclusion criteria. Individuals who met the clinical criteria but declined participation were excluded. Information about the study was posted on an information board in the outpatient clinic area designated for the target group. A research assistant screened and identified eligible participants based on the predefined criteria, resulting in a total of 14 patients who participated in the study.

Eligibility criteria included having firsthand experience relevant to the phenomenon under investigation, the ability to communicate in either standard Thai or Northeastern Thai, and a willingness to participate. Interviews were arranged at times and locations convenient for participants. Data collection continued until phenomenological saturation was achieved, defined as the point at which no new meanings or insights emerged; saturation was reached after 14 interviews.

### Data Collection

Data were gathered through in-depth phenomenological interviews using open-ended questions designed to elicit participants’ experiences. Interview prompts included, “Please describe your experiences of being overweight, from the time you first perceived your body as larger than others to your decision to undergo gastric bypass surgery,” “How did this situation make you feel?,” and “What meaning does this experience have for you?”

All interviews were conducted by the same research assistant to ensure consistency. Prior to each interview, participants were informed of their rights, ethical considerations, and the voluntary nature of their participation. Participants then completed a brief demographic information form, and consent was obtained to audio-record the interviews. Each interview lasted approximately 60‐90 minutes. Following each session, the research assistant documented field notes capturing contextual details, emotional expressions, pauses, and nonverbal cues. Audio recordings were transcribed verbatim within 18 hours of the interviews.

### Data Analysis

Data analysis was conducted independently by researchers 1 and 2 at the initial stage. Their analytic outputs were subsequently subjected to peer debriefing and discussed collaboratively with the research team and research assistants to achieve consensus. The analysis adhered to Colaizzi's 7-step phenomenological approach [20]: (1) immersion in participants’ narratives, (2) identification of significant statements, (3) formulation of meanings, (4) clustering of meanings into themes, (5) development of textural and structural descriptions, (6) synthesis of the fundamental structure of the phenomenon, and (7) validation of findings through member checking with participants. In case of coding disagreement, systematic consensus discussion with peer debriefing was performed to finalize the coding based on the raw data.

### Trustworthiness and Rigor

Methodological rigor was established in accordance with the criteria proposed by Lincoln and Guba and was reported following COREQ items 28‐32 [21-23]. Data saturation was achieved through iterative and concurrent data collection and analysis. Themes were inductively generated using the Colaizzi analytic framework. Credibility was strengthened through member checking, phenomenological validation, and peer debriefing. Dependability and confirmability were supported through the maintenance of an audit trail, the use of reflexive journals, systematic bracketing, and the inclusion of verbatim participant quotations. Transferability was enhanced by providing in-depth descriptions of the study context and participant characteristics. All themes were clearly delineated in the findings.

### Ethical Considerations

Ethical approval for the study was obtained from the Human Research Ethics Committee of Khon Kaen University, Thailand (approval no. HE651187). Written and/or verbal informed consent was obtained from all participants prior to data collection. Confidentiality and voluntary participation were ensured throughout the study. To protect participants’ identities, pseudonymous identification codes (eg, ID1 and ID2) were used in place of real names. Each participant received compensation of 300 Thai baht (approximately US $10).

## Results

### Participant Characteristics

Fourteen patients met the study criteria and agreed to participate in the study; no patients declined to participate in the study. Baseline characteristics of the patients are shown in Table 1. The average age of all patients was 27.21 (SD 4.98) years. The study included 28.57% (4/14) males; 78.57% (11/14) of all participants had a single marital status. Most patients had a monthly income of US $333 or more (13/14, 92.86%). Four patients had no comorbidities (4/14, 28.57%), and the other 4 patients had hypertension (4/14, 28.57%). Half of all patients (7/14, 50%) had basic universal insurance coverage. The average BMI was 45.28 (SD 7.58) kg/m^2^ and an average AHI of 40.42 (SD 29.61) events per hour, indicating severe OSA was reported. All participants were employed, while 1 patient was working and in school. This study was conducted in northeastern Thailand, where the residential culture included large families comprised of 2 or 3 generations. The cultural beliefs for the family included taking care of the younger generations, as problems of the younger generations were considered family problems.

**Table 1.** Baseline characteristics and polysomnography results in adult patients with obstructive sleep apnea who planned for bariatric surgery.

Factors	Results
Age (years), mean (SD)	27.21 (4.98)
Sex (male), n (%)	4 (28.57)
Education, n (%)	
Under Bachelor	3 (21.43)
Bachelor	11 (78.57)
Marital status, n (%)	
Single	11 (78.57)
Married or separated	3 (21.43)
Occupation, n (%)	
Government officers	6 (42.86)
Business	6 (42.86)
Freelance	1 (7.14)
Student	1 (7.14)
Income, US $/month, n (%)	
< US $333/month	1 (7.14)
≥US $333/month	13 (92.86)
Comorbid diseases, n (%)	
No	4 (28.57)
Hypertension	4 (28.57)
Diabetes	1 (7.14)
Fatty liver	1 (7.14)
Polycystic ovarian disease	3 (21.43)
Allergic rhinitis	1 (7.14)
Smoking	0
Alcohol consumption	9 (64.29)
Insurance, n (%)	
Government	5 (35.71)
Social Security Scheme	2 (14.29)
Basic universal coverage	7 (50.00)
Epworth Sleepiness Scale, mean (SD)	8.07 (2.75)
STOP-Bang score^a^, mean (SD)	4.42 (1.22)
BMI (kg/m^2^), mean (SD)	45.28 (7.58)
Neck circumference (cm), mean (SD)	41.14 (5.45)
Apnea-hypopnea index (events/h), mean (SD)	40.42 (29.61)

a STOP-Bang score: snoring, tiredness, observed apnea, hypertension, BMI, age, neck circumference, gender.

### Perspectives of Adult Patients With OSA Who Planned for Bariatric Surgery

Qualitative research on the perspectives of young adults regarding gastric bypass surgery for weight loss due to severe obesity was conducted. The results explain the factors and conditions that influenced their approach to solving this problem and conclude with recommendations for best practices for similar situations in other contexts (Tables2  3).

**Table 2.** Examples of Colaizzi code-to-theme in adult patients with OSA^a^ who planned for bariatric surgery.

Significant statements (raw data)	Formulated meanings (interpretation)	Cluster of meanings or subthemes	Emerging themes
“I don’t care about weight; it does not matter to me, despite being bullied at school.”	The digital era with its unlimited connections among people and devices may lead to a sedentary lifestyle of the younger generation.	Stuck in one’s own private world	Love of eating leads to critical obesity
“I believed that my parents were uncomfortable too, but did not want to blame it on their children.”	Protect your children from the ridicule of society.	Parents try to isolate their children from society, so they do not have to endure bullying.	Obese persons: strangers in society
“I was always bullied, and this made me ashamed, particularly in public transportation.” “You are so big, you take more space; you take two seats in the public transportation. I cannot sit beside you. I was only just big sized.”	Feeling ashamed, uncomfortable, and worried about their social appearance.	Never chosen to be a school representative by classmates or teachers	Obese persons: a stigma from society as incompetent
“Being obese, I have lost opportunities for jobs, as employers may think that I am not active or not flexible and not suitable for the work”	Being insecure about their future, jobs, economic security, and social acceptance.	Obesity leading to lost opportunities in life	Obesity and opportunity for job
“It was good to chat with the group. I chatted with others, as they had done that before me. For example, [they discussed] how to refer themselves to be treated with bariatric surgery at this hospital. It was a page called ‘bariatric surgery’ at University Hospital.”	Online communities serve as platforms for sharing experiences and buying or selling inexpensive used CPAP^b^ machines.	The benefits of joining groups on social media	Social media on weight loss

a OSA: obstructive sleep apnea.

b CPAP: continuous positive airway pressure.

**Table 3.** Summary of themes of a qualitative study in adult patients with OSA^a^ who planned for bariatric surgery.

Main themes and themes	Descriptions
Main theme 1: obesity-related themes (4 themes)	
Eating behaviors and an enduring attachment to food as pathways to obesity	Obesity results from childhood development where parents and families use eating as a way to show affection, rather than encouraging physical activity. Furthermore, Thai society has a value system where overweight children are affectionately referred to as “chubby.”
Experiencing social othering and stigmatization as a person with obesity	Overweight people feel alienated from society, uncomfortable in public, and have to endure hurtful questions about their weight. Their parents likely feel the same way, but do not say anything for fear of upsetting their children.
Perceived loss of life opportunities, particularly in employment, associated with obesity	Being overweight often leads to social stigmatization, making students feel incompetent, overlooked, and insignificant. They are rarely nominated to represent their school in any activities.
The turning point: making a transformative decision to escape the condition of obesity	Obesity can lead to a lack of confidence, making people hesitant to apply for competitive jobs and preferring less competitive work, while overweight individuals desire self-reliance and the ability to stand on their own 2 feet in society.
Main theme 2: weight loss experience (2 themes)	
The lived experience of CPAP^b^ use as a transformative life passage	When overweight individuals reach a point where they can no longer tolerate it, they decide to seek various weight-loss methods, but without success. They may lose weight temporarily, but then regain it.
Social media on weight loss	In seeking ways to lose weight, they found diverse groups of friends online, which led them to decide to undergo gastric surgery.
Main theme 3: CPAP therapy prior to bariatric surgery (1 theme)	
CPAP purchasing and bariatric surgery from online platforms	Preparation for gastric surgery requires checking for sleep apnea, which is common in obese individuals and often necessitates the use of CPAP for 1 month prior to surgery. Information on social media regarding gastric surgery for weight loss contains both useful information and misconceptions that do not align with scientific data. Furthermore, many parties use this information as a source of recruitment and income for their health care businesses.

a OSA: obstructive sleep apnea.

b CPAP: continuous positive airway pressure.

There were 3 main themes, including obesity-related themes (4 themes), weight loss experience (2 themes), and CPAP therapy prior to bariatric surgery (1 theme). Details of each main theme and its themes are as follows:

### Main Theme 1: Obesity-Related Themes (4 Themes)

#### Theme 1: Eating Behaviors and an Enduring Attachment to Food as Pathways to Obesity

The characteristics of informants included being a single child or 1 of 2 children in the family. The eating habits and love of eating began when they were children, leading to childhood obesity. Family members were proud to have a lovely, obese child. Patients developed the habit of overeating and never stopped eating particularly sweets, fried foods, and soft drinks. Parents allowed their children to overeat and never told them not to eat as they felt that their children were happy when they ate.

The digital era, with its unlimited connections among people and devices such as smartphones, tablets, and laptops, may result in a sedentary lifestyle for the young generation. Sitting, eating, and interacting with digital devices after school does not include regular exercise, which was part of the daily routine of the patients when they were children. The patients indicated that watching online content and consuming snacks and soft drinks after school were their happiest school-age moments. They did not like to exercise, claiming it was hot outside and they became tired, sweaty, and itchy.

Parents were aware of and unhappy about their children’s growth. They tried to find some solutions for weight reduction, which was unknown to their children. The patients claimed not to care about weight, despite being bullied by classmates.

I eat a lot, maybe two of three times that of my friends. I eat three big meals a day, and I have been obese since I was a primary school child. I was bullied as an obese kid.

[ID06, male, 26 years, single, 113 kg]

I was a big kid since I was in primary school, but I did not feel anything was wrong with my body. But, my parents were afraid that I may have a health problem in the future, and they wanted me to lose weight. as they were really concerned about my life.

[ID10, male, 27 years, single, 150 kg]

When I was a child, I ate a lot. My parents took my food away, but I loved to eat.

[ID14, female, 30 years, single, 128 kg]

#### Theme 2: Experiencing Social Othering and Stigmatization as a Person With Obesity

Participants in this study did not use the word “obese” but used “big sized or overweight.” They reported that they felt uncomfortable in society when someone asked their parents why their kid was obese. They also asked, “How much do you weigh?” They believed that their parents were uncomfortable too, but they did not want to blame their children.

I was a big sized kid and friends always bullied me by calling me ‘obese kid’ and ‘disgusting.’ They asked me, ‘Why are you so obese?’ I was very upset with these situations. I did not disturb anyone, but they bullied me about my body. Sometimes, I would cry and feel upset that I had a bad shape unlike others and why I had to be big sized.

[ID14, female, 30 years, single, 128 kg]

#### Theme 3: Perceived Loss of Life Opportunities, Particularly in Employment, Associated With Obesity

The worst scenarios for obese people occur in school. Obese kids in school were never chosen to be a school representative by classmates or teachers. This resulted in the social isolation of obese students. They did not attend school social events and felt disappointed for not being invited by others to attend these events.

Those who were obese also felt ashamed, uncomfortable, and worried about their social appearance as they were considered attractive as children. Children innocently asked their parents why this guy was so obese and pointed to them in public areas. Additionally, some people made obese individuals uncomfortable on public transportation as they were concerned that they took up more space for seating.

I was bullied about my body from children, adults, or even elderly people. I was very upset, as I did not want to be obese.”

[ID02, female, 36 years, married with two children, 127 kg]

I was always bullied, and this made me ashamed, particularly in public transportation. ‘You are so big, you take more space; you take two seats in the public transportation. I cannot sit beside you.’ I was only just big sized.

[ID04, female, 33 years, married with one child, 120 kg]

My neighbors bullied me, asking how I got a job or passed the exam. They thought that I did not do anything other than eat and sleep.

[ID05, female, 26 years, single, 115 kg]

I want to be healthy and active, but I could not lose weight despite several tries. I am not confident with my body shape when speaking in public. Additionally, I feel easily tired and get headaches.

[ID06, male, 26 years, single, 113 kg]

Being big sized made me insecure, and I felt unvaluable. Sometimes, I thought, ‘Why am I so strange and unlike others?’ I want to be good looking and get compliments from others.

[ID14, female, 30 years, single, 128 kg]

#### Theme 4: The Turning Point: Making a Transformative Decision to Escape the Condition of Obesity

Most patients had a job that was not competitive, such as working with family members or previous mentors. Participants felt a lack of confidence about their future, employment, economic security, and social acceptance. The main need of participants was social acceptance for obese people. Being obese is a repetitive social stigma with a significant impact, resulting in low self-esteem. How society interacts with and perceives obese people is a critical issue in the current era.

Being obese, I have lost opportunities for jobs, as employers may think that I am not active or not flexible and not suitable for the work.

[ID10, male, 27 years, single, 150 kg]

I may not work online forever. I hope that I can lose weight after bariatric surgery and that I may make myself healthier. I want to change my job and apply for a new one. If I am confident, I may get a good job.

[ID13, female, 24 years, single, 110 kg]

After college, I did not get a job for one year. I decided to work with my family at a bakery.

[ID06, male, 26 years, single, 113 kg]

### Main Theme 2: Weight Loss Experience (2 Themes)

#### Theme 5: The Lived Experience of CPAP Use as a Transformative Life Passage

Participants reported several strategies for losing weight, which included diet control, exercise, and medication. They learned these strategies from various sources, such as family members, friends, the internet, and health care personnel. Participants were able to lose some weight with support from family members, but they gained the weight back and experienced yo-yo effects.

I tried low-carb diets and intermittent fasting, but it was difficult. I also used to attend the weight loss project at Herbalife for two months. Then, they tried to sell me some Herbalife products. As the products were so expensive, I quit the program.

[ID02, female, 36 years, married with two children, 127 kg]

I have tried several methods, but they did not work. I used to fast and did not eat dinner, but I was so hungry. I was able to do that for ten days. Then, I tried to exercise but it resulted in ankle pain which occurred because of obesity. My doctor told me to walk instead. I also ate keto diets but I got a rash and syncope from keto diets. I tried keto diets for 15 days, but then I quit.

[ID04, female, 33 years, married with one child, 120 kg]

I was bullied about my weight, so I tried to lose weight using over-the-counter medications and several other methods. I was able to lose some weight, but I gained weight back after I stopped taking the medication. I felt hungrier after quitting the medications. While I took them, I felt palpitations. I saw some news about the dangers of the medications; some people had died due to weight loss medications. My mom gave me some medication but I did not take them.

[ID05, female, 26 years, single, 115 kg]

I tried to lose weight by diet control and intermittent exercise. But I felt fatigue and dyspnea from exercise. So, I quit exercise.

[ID06, male, 26 years, single, 113 kg]

I tried to lose weight due to ankle pain. I could not stand when I woke up or after long sitting as it caused ankle pain.

[ID07, female, 25 years, single, 175 kg]

I had to take medications for losing weight. If I took medication, my weight dropped to 150 kg. I weighed 170 kg or more if I did not take medications. I was afraid of weight loss medications.

[ID08, male, 27 years, single, 150 kg]

I did not eat carbs or sugar. I lost 11 kg, but I gained weight after I stopped doing that. I could not sleep if I did not eat dinner, as I was hungry.

[ID10, male, 27 years, single, 150 kg]

I was so lazy about losing weight. I was able to control my diet and do more exercise, but I did that for only a short period of time.

[ID11, female, 19 years, single, 90 kg]

I have tried several medications since I was 20 years. If I stopped medications, I gained the weight back. There were several medications advertised on Facebook.

[ID12, female, 29 years, single, 95 kg]

I tried several methods but they did not work as I worked on a shift. I was not healthy and felt dizzy while exercising. So, I decided to do surgery.

[ID13, female, 24 years, single, 110 kg]

My highest weight was 130 kg. I lost 26 kg by eating a keto diet and exercising. My weight gained back to 130 kg after eating as usual.

[ID14, female, 30 years, single, 128 kg]

#### Theme 6: Social Media on Weight Loss

The study participants were young adults with social stigma, which caused social isolation. Using digital media, these participants have access to content in such media, which includes several available methods for losing weight, including bariatric surgery. Searching on Facebook (Meta Platforms, Inc.) using the keywords “lose weight,” there were at least 50 groups with more than 24,000 members. Other platforms also have media on weight loss, including TikTok (ByteDance Ltd) and YouTube (Google LLC). These media were used for sharing experiences and receiving support from others. There were several hidden services in these platforms, such as multidisciplinary services for weight reduction.

I like these groups on Facebook, as I had someone to talk with regarding bariatric surgery, as it was so dangerous. My husband suggested I do bariatric surgery, as he was afraid that I may have a stroke like my aunt. I got a lot of useful information on bariatric surgery from these groups. It was a good platform for idea exchange as well as experience exchange on bariatric surgery such as post-operative pain.

[ID04, female, 33 years, married with one child, 120 kg]

It was good to chat with the group. I chatted with others, as they had done that before me. For example, [they discussed] how to refer themselves to be treated with bariatric surgery at this hospital. It was a page called ‘bariatric surgery’ at University Hospital.

[ID13, female, 24 years, single, 110 kg]

I was on TikTok but never thought about going to a page on bariatric surgery. I had a lot of followers on TikTok after I accessed that page. I told them every day about my symptoms after the bariatric surgery. Some patients who had had the bariatric surgery also shared their experiences on my page while those who planned to have the bariatric surgery would have ideas on it.

[ID14, female, 30 years, single, 128 kg]

I found this weight loss clinic on TikTok, which popped up. The page reviewed bariatric surgery, how much weight reduction after surgery, and other contents. I never thought that I needed the bariatric surgery, but I was interested in doing the surgery after learning from that page on TikTok. I felt tired easily and unable to live normally, maybe from obesity. I decided to go for surgery, as it only took a few days to recover from bariatric surgery, which was mentioned on the page.

[ID01, female, 34 years, single, 118 kg]

I found several pages on weight loss posted by several private hospitals. I found that many people underwent bariatric surgery. I did a study on bariatric surgery and found that it could result in a lot of weight reduction. I was unsure if I would go for the surgery. So, I did a study again and found that undergoing the bariatric surgery at the government hospital was a lot cheaper than the private hospital. I learned a lot from the weight loss pages on online media.

[ID14, female, 30 years, single, 128 kg]

### Main Theme 3: CPAP Purchasing Prior to Bariatric Surgery (1 Theme)

#### Theme 7: CPAP Purchasing and Bariatric Surgery From Online Platforms

Patients who planned to undergo bariatric surgery needed to be tested for OSA. Those with OSA required treatment with a CPAP machine for at least 1 month prior to the surgery. Patients in this study had an AHI between 12 and 100 events per hour. Even though some patients had a very high AHI, they were not concerned about OSA. They believed that OSA would be cured after the bariatric surgery, as their weight would be reduced. Therefore, CPAP treatment was temporary for these patients and not a lifesaving machine. Seven patients decided to buy the CPAP or a secondhand CPAP, while the other 7 patients decided to rent the CPAP for 1 month instead of purchasing it. For those who purchased the equipment, 3 patients (42.86%) had insurance coverage, and 2 patients (28.57%) who rented the CPAP also had coverage. In some circumstances, CPAP for rent was not available. Therefore, some patients looked for secondhand CPAP online using several platforms.

I had severe OSA with an AHI of 44 events/hour. I had to use a CPAP but it was so expensive. I did research online and found that my snoring and OSA would disappear if I lost weight after the bariatric surgery. I did not want to buy the CPAP, and chose to rent one instead for one month.

[ID14, female, 30 years, single, 128 kg]

Prior to bariatric surgery, my doctor told me to buy a CPAP and use it before the surgery. But, it was expensive and I chose to rent it. At that time, CPAP for rent was not available. I searched for CPAP online from Google and found a second-hand CPAP on Facebook. It was cheaper at US$ 266.

[ID02, female, 36 years, married with two children, 127 kg]

Online platforms have both advantages and disadvantages regarding bariatric surgery. The main advantages include sharing data before and after surgery, sharing information regarding used CPAP machines, working as a case finder for bariatric surgery, and psychosocial support. However, data on the online platforms may not be written by health professionals, and some information may be incorrect and misleading. Some people may have advantages from case finding or selling CPAP online for preoperative use.

### Benefits

Individuals who underwent bariatric surgery may have different symptoms and responses to the surgery. I read from the online content and used that as a guide for our preparation. I did not feel lonely when searching or chatting on the online platforms. I felt like I had friends taking care of me. It is an experience sharing in the online platforms.

[ID13, female, 24 years, single, 110 kg]

Online information originates from anywhere in the country. Some people on the platforms acted like coordinators for bariatric surgery. They took care of patients who were willing to perform the surgery every step of the way, such as checking for the surgery queue, providing preoperative CPAP equipment, checking for insurance, or even taking care of the patients during the operative and postoperative periods. These were occupations for bariatric surgery. CPAP in these teams were sold to anyone as second-hand CPAP.

[ID04, female, 33 years, married with one child, 120 kg]

### Disadvantages

Reading online content too much made me anxious and worried, such as severe vomiting after bariatric surgery, severe pain at the surgical site, or infected surgical wounds. Some information may not be correctly posted, as they are not doctors.

[ID12, female, 29 years, single, 95 kg]

## Discussion

This qualitative study has shown causes of obesity, effects of obesity, experiences of weight loss and social media on weight loss, CPAP therapy prior to bariatric surgery, and social media regarding bariatric surgery. Social media was used mainly for weight loss and bariatric surgery preparation. Patients did not seek the benefits of CPAP therapy prior to the surgery.

This study found that morbidly obese patients who planned to undergo bariatric surgery showed that their condition caused several personal and social limitations. As shown in Themes 2‐4, these patients were socially stigmatized and lost job opportunities. These findings were compatible with a previous qualitative study from the United Kingdom regarding shame and stigmatization [24]. This study added that obesity was mainly due to personal eating habits and/or parental responses. The results showed that a love of food was the main cause of obesity. Additionally, their parents did not prevent them from eating, which might be another cause of childhood obesity.

Even though patients tried several strategies to lose weight (Theme 5), they were unable to maintain a low body weight. These attempts resulted in seeking rapid weight loss or bariatric surgery. As previously reported, bariatric surgery is the most effective means for morbidly obese patients [25]. However, previous studies showed that data or resources were limited in terms of support groups or teen-friendly resources [12,13]. This study found that there were several support groups, resources, or information regarding both weight reduction and bariatric surgery on platforms, such as Facebook or YouTube. These social media sites are easily accessible and suitable for young adults to gain information. The two main limitations of these platforms were (1) data may not be valid as some pages or groups are not run by health care providers, and (2) some pages may benefit by recruiting patients to their health care systems. These may be employees who receive payments from the health care systems.

“CPAP machine is not a lifesaving machine and may not be required after bariatric surgery.” (ID02) Even though several studies have shown the benefits of CPAP therapy on cardiovascular outcomes [26-28], morbidly obese patients who planned to undergo bariatric surgery did not believe that CPAP was a lifesaving machine; rather, it is a temporary treatment. Additionally, these patients did not plan to use a CPAP machine after the bariatric surgery. However, data have indicated that OSA still persisted after bariatric surgery. A meta-analysis found that the average AHI was decreased from 40.3 to 13.5 events per hour after bariatric surgery, which indicated that CPAP was still required [29]. The patients were not aware of this, even those who purchased the CPAP for use prior to the study. Like ID02, they may sell the CPAP machine as a secondhand machine for other patients to use for a month prior to bariatric surgery. Some patients believed that bariatric surgery offers hope to cure obesity as well as OSA [24,25].

These results may indicate that marketing strategies are required for morbidly obese patients who plan to undergo bariatric surgery. These include social media platforms for bariatric surgery information as well as customer packages for bariatric surgery. Such packages may include options of hospitals in various areas in Thailand, the cost of bariatric surgery at each hospital, and surgery options. This could be a new online occupation [25]. Another marketing strategy would be to use social media for bariatric surgery preparation, advertising such needs as secondhand CPAP or CPAP rentals. In addition, social media could provide support groups for patients who plan to undergo bariatric surgery. Such groups would consist of a multidisciplinary team including surgeons, nutritionists, sleep physicians, and psychiatrists or psychologists [13,24,25].

As previously reported, qualitative studies showed that online health information was the main source and had an increasing trend of using online or internet [30-32]. However, there were some barriers to online health information, including limited eHealth literacy or inconsistency of online information [32]. A systematic review also reported findings from qualitative studies showing that online health information was reliable in only 40% and 60% of health information consumers searched at least 3 different websites [33]. In this study, participants searched for data from social media and believed in the data. Therefore, the results of the study may not be correct based on clinical studies, but indicate the participants’ beliefs. The previous qualitative study also suggested that the online platforms created by health care professionals may be more reliable and can be used for medical health decisions. However, individualized medical decision-making may be needed and crucial [34,35].

There are some limitations in this study. Most participants were young adults who were mainly single and female. These data aligned with previous studies indicating that female, young adult patients are concerned about their physical images [24,25]. However, data in this study were collected until data were saturated. Additionally, no intervention was applied in this study.

In conclusion, morbidly obese patients with OSA who were planning bariatric surgery experienced physical and mental challenges related to obesity. These patients had failed various other weight loss programs and believed that bariatric surgery was the correct solution. Social media platforms are sources of information prior to bariatric surgery. A CPAP machine is not a lifesaving machine but just a temporary treatment before surgery and may not be required after weight loss. CPAP rental or secondhand purchasing may be preferred. These are participant beliefs, not clinical conclusions.

## Abbreviations

AHI: apnea-hypopnea index
COREQ: Consolidated Criteria for Reporting Qualitative Research
CPAP: continuous positive airway pressure
OSA: obstructive sleep apnea
